# Outbreak of recombinant lumpy skin disease virus in yaks: high mortality and systemic pathogenesis in Qinghai-Tibet Plateau yak herds

**DOI:** 10.3389/fvets.2025.1584975

**Published:** 2025-05-21

**Authors:** Jianwu Hu, Yan Li, Xiaohu Zhang, Yaozhong Lu, Zhibo Zeng, Mengen Xu, Siyang Mou, Shah Nawaz, Dongjing Wang, Tianwu An, Xiaowei Li, Quan Mo, Jiakui Li

**Affiliations:** ^1^College of Veterinary Medicine, Huazhong Agricultural University, Wuhan, China; ^2^Department of Agriculture and Rural Affairs of Tibet, Lhasa, China; ^3^Institute of Animal Husbandry and Veterinary, Tibet Autonomous Region Academy of Agricultural Sciences, Lhasa, China; ^4^Sichuan Academy of Grassland Science, Chengdu, China; ^5^Longri Livestock Breeding Farm, Aba Tibetan and Qiang Autonomous Prefecture, China; ^6^College of Animals Husbandry and Veterinary Medicine, Tibet Agricultural and Animal Husbandry University, Linzhi, China

**Keywords:** LSDV, recombinant strain, Qinghai-Tibet Plateau, yak, prevention and control

## Abstract

The emergence of recombinant *Lumpy Skin Disease Virus* (LSDV) strains in Asia has led to outbreaks marked by severe skin nodules, high transmissibility, and transboundary spread, resulting in significant economic losses to cattle industries in China and neighboring countries. The Qinghai-Tibet Plateau, historically a natural barrier against viral incursions, has recently experienced increasing LSDV cases in yaks (*Bos grunniens*). Current study elucidates the threat posed by recombinant LSDV strains to yaks through clinical, pathological, and molecular analyses. Field observations revealed infected yaks exhibited fever, dyspnea, cutaneous pox lesions, lymphadenopathy, and mucosal lesions. Viral DNA detection showed 100% positivity in skin samples (6/6), 53.33% (8/15) in nasal swabs, and 33.33% (5/15) in anal swabs, with an overall mortality rate of 46.67% (7/15). Necropsy identified respiratory and digestive system lesions, including tracheal congestion, pulmonary hemorrhagic plaques, and ruminal serosal hemorrhagic masses. Histopathology demonstrated dermal vasculitis, lymphocytic infiltration, and viral inclusion bodies. Immunohistochemistry localized viral antigens to hair follicle epithelia and macrophages. Phylogenetic analysis positioned the yak-derived LSDV strain (LSDV/China/GS/Yak) within the Cluster 1.2 recombinant subclade with high homology to recombinant strains circulating in East/Southeast Asia but differing from non-recombinant Indian Cluster 1.2 strains. The results emphasize increased pathogenicity of recombinant LSDV in plateau yaks and convey the critical need for region-specific control strategies.

## 1 Introduction

Lumpy skin disease (LSD) is a transboundary infectious disease caused by the lumpy skin disease virus (LSDV), primarily transmitted by insect vectors. It affects cattle including Water buffalo (*Bubalus bubalis*), giraffes (*Giraffa camelopardalis*), impalas (*Aepyceros melampus*), Arabian oryx (*Oryx leucoryx*), and Indian gazelles (*Gazella bennettii*), among other species (1–6). In cattle, LSD is characterized by cutaneous nodules, fever, and systemic infection, leading to reduced milk production, compromised reproductive efficacy in bulls, and economic losses in beef cattle (7, 8).

LSDV is a member of the genus *Capripoxvirus* within the subfamily chordopoxvirinae (family poxviridae), along with the sheeppox virus (SPPV) and goatpox virus (GTPV) (9, 10). It is an enveloped, double-stranded DNA virus with a 151 kb genome, sharing over 95% nucleotide homology with SPPV and GTPV, and exhibiting cross-immunogenicity among *capripoxviruses* (11). Current commercial vaccines against LSDV include homologous vaccines (attenuated Neethling field strains) and heterologous vaccines (live-attenuated GTPV and SPPV) (1). Its vaccination is very effective and provides potent protection from specific pathogens, but noteworthy is that mild “Neethling-like” signs can appear after immunization. While generally benign and harmless, there is valid reason to have reservations concerning the safety profile of live-attenuated immunization overall. It is therefore essential in this context to closely monitor the situation, followed by thorough investigation to improve immunization strategies in the future (1, 12).

Historically, LSD was endemic to sub-Saharan Africa until the 1990s (12, 13). The first African outbreaks outside the continent were reported in the Middle East in 1989, followed by sporadic cases (14–16). By 2014, LSD had spread to Balkan countries (17–19). In 2017, a vaccine-escape recombinant LSDV strain—derived from genetic recombination between Neethling-like vaccine strains and field isolates—was identified in Russia near the Kazakhstan border (20). Over the past decade, LSDV has expanded into Europe and Asia, now circulating in East, South, and Southeast Asia (21, 22). Notably, two distinct LSDV lineages co-circulate in Asia: (1) recombinant vaccine-like strains (e.g., in China, Mongolia, Thailand, and Vietnam) and (2) classical field strains (e.g., in Bangladesh, India, Nepal, and Myanmar) (18, 23–29). This dual viral pressure highlights the ongoing threat to Asian cattle populations.

Yaks (*Bos grunniens*), a cold-adapted ruminant species with a global population exceeding 20 million, are predominantly (>95%) distributed across the high-altitude (3,000–5,000 m), hypoxic regions of China’s Qinghai-Tibet Plateau (30, 31). Dubbed the “ship of the plateau,” yaks serve as a critical livestock resource, providing meat, hides, and dairy products for local communities (32). LSDV outbreaks have inflicted severe economic losses on global cattle industries (8). Given the harsh environmental conditions and limited veterinary infrastructure in yak habitats, the introduction of LSDV presents a catastrophic risk to yak farming on the plateau. The correspondence of heightened susceptibility among yak populations and the high transmissibility of recombinant LSDV strains requires in-depth understanding of viral pathogenesis in this novel host species. This study investigates the clinicopathological features and molecular epidemiology of LSDV infections in Qinghai-Tibet Plateau yaks, underscoring the escalating threat to high-altitude ecosystems and the urgent need for cross-border surveillance. Longitudinal research involving next-generation molecular tools, including whole-genome sequencing and phylogenetic analysis, is essential to track viral evolution and the development of new recombinant strains to implement preventive disease management and control strategies in this vulnerable ecosystem.

## 2 Materials and methods

### 2.1 Sample collection and viral DNA extraction

In 2023, samples were collected from 15 yaks (*Bos grunniens*) exhibiting cutaneous nodules or pox-like lesions at a farm in Gannan Tibetan Autonomous Prefecture, China, suspected of being infected with LSDV. Samples included blood, nasal and anal swabs (15 from each animal), and 6 skin tissues. Nasal and anal swabs were immediately placed in 800 μL phosphate-buffered saline (PBS), while blood specimens were permitted to stay at room temperature for 2 h to obtain serum and were subsequently centrifuged at 6,000 rpm for 5 min to discard coagulated red cells. Specimens were shipped to the laboratory under cold chain conditions to −20°C and were preserved at −80°C until further handling.

Approximately 1 g of skin tissue was homogenized in 1 mL PBS using an Automatic Sample Fast Grinder (Jingxin, Shanghai, China). The homogenate, along with other samples, was centrifuged at 9,000 rpm for 3 min at 4°C. Viral DNA was extracted from 400 μL of each sample using the VAMNE Magnetic Pathogen DNA/RNA Kit (Prepackaged) (Nanjing Vazyme, Nanjing, China) following the manufacturer’s protocol.

### 2.2 qPCR detection of LSDV DNA

LSDV DNA was detected using quantitative polymerase chain reaction (qPCR) with primers targeting the GPCR gene (19, 33), modified as follows: Forward primer: 5′-agtcgaatataaagtaatcagtc-3′, Reverse primer: 5′-ccgcata-taatacaacttattatag-3′. Each 20 μL reaction contained: 10 μL Taq Pro Universal SYBR qPCR Master Mix (Nanjing Vazyme, Nanjing, China), 0.4 μL each of forward and reverse primers (10 μM), 2 μL template DNA, 7.2 μL distilled H₂O. Thermal cycling conditions: 95°C for 30 s (pre-denaturation), 40 cycles of 95°C for 5 s and 60°C for 30 S. Melt curve analysis was performed using the default settings. All reactions were performed on a LightCycler^®^ 96 Instrument (Roche Diagnostics, Basel, Switzerland) Melt curve analysis was performed using the default settings.

### 2.3 Histopathology and immunohistochemistry analysis

Fresh nodule tissues were fixed in 10% neutral buffered formalin, washed with PBS, dehydrated through graded ethanol (70, 95, 100%), cleared in xylene, embedded in paraffin, and sectioned at 3 μm. Sections were stained with hematoxylin and eosin (H&E) for histopathological analysis. For immunohistochemistry (IHC), adjacent sections were incubated with mouse anti-LSDV126 protein monoclonal antibody (generously provided by Prof. Yuefeng Sun, Lanzhou Veterinary Research Institute) following established protocols (34). Slides were scanned using an automated slide scanner (3DHistech, Budapest, Hungary) and visualized with SlideViewer v2.7.^1^

### 2.4 Whole-genome sequencing

The whole genome sequencing was performed on the Illumina NovaSeq 6,000 PE150 platform. Raw reads were quality-controlled using Trimmomatic v0.39 (35), and clean reads were mapped to the LSDV reference genome (China/GD01/2020; GenBank: MW355944.1) using Bowtie2 v2.5.2 (36). Contigs were assembled using MEGAHIT v1.2.9 (37) and further polished with SeqMan module in DNASTAR Lasergene v11^2^ to generate complete genomes. We have submitted the genomic sequences to the GenBank database, GenBank No. MW355944.1.

### 2.5 Phylogenetic analysis

Full-genome, GPCR, and RPO30 sequences of LSDV, sheeppox virus (SPPV), and *goatpox virus* (GTPV) were retrieved from the NCBI database.^3^ Sequence alignment was performed using MAFFT v7.505 (38) with the FFT-NS-1 (fast) strategy and default alignment parameters within the PhyloSuite v1.2.3 software package (39). The best substitution model was selected via ModelFinder v2.2.0 (BIC criterion) (40). Maximum likelihood trees were constructed using IQ-TREE v2.2.0 (1,000 bootstrap replicates) (41) and visualized using Chiplot^4^ (42).

## 3 Results

### 3.1 Field observations

**Figure 1:**
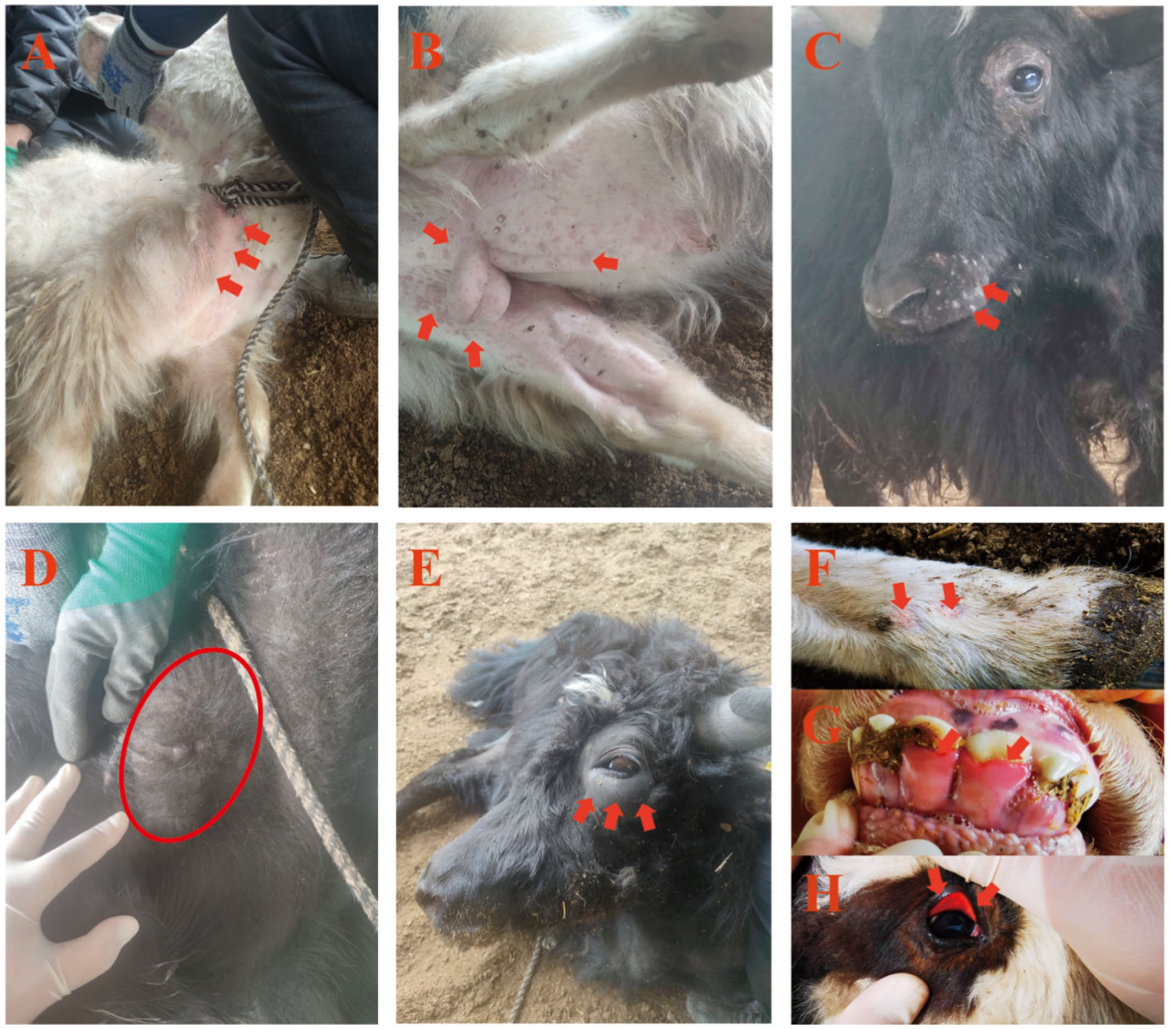
Clinical manifestations of LSDV infection in yaks. **(A–C)** Cutaneous pox nodules; **(D)** Enlarged superficial cervical lymph nodes; **(E)** Periorbital skin edema; **(F–H)** Pox lesions on hooves, gingival swelling, and conjunctival hyperemia.

Observed clinical signs of LSDV infection of yaks were tachypnea, haemorrhagic diarrhea, fever, and skin lesions of characteristic pox-like appearance. The lesions were most prominent on hairless or lightly covered skin surfaces, i.e., nasal planum, ventral cervical region, perineum, and the inguinal region (Figures 1A–C). Initial lesions manifested as erythematous skin patches (Figure 1A), progressing to raised nodules (Figure 1B), and eventually forming scabs that shed, leaving scars (Figure 1C). Enlarged, firm superficial cervical lymph nodes (lymphadenopathy) were observed in some yaks (Figure 1D). Soft periocular swelling (Figure 1E), hoof lesions, gingival mucosal erythema, and conjunctival hyperemia were noted in individual cases (Figures 1F–H).

### 3.2 Viral detection and necropsy findings

Samples were collected from 15 yaks displaying typical LSDV symptoms. qPCR detected LSDV DNA in 53.33% (8/15) of nasal swabs, 33.33% (5/15) of anal swabs, and 100% (6/6) of skin tissues. Seven yaks with severe clinical signs died, resulting in a mortality rate of 46.67% (7/15) while the morbidity rate was recorded as 100% (15/15).

**Figure 2:**
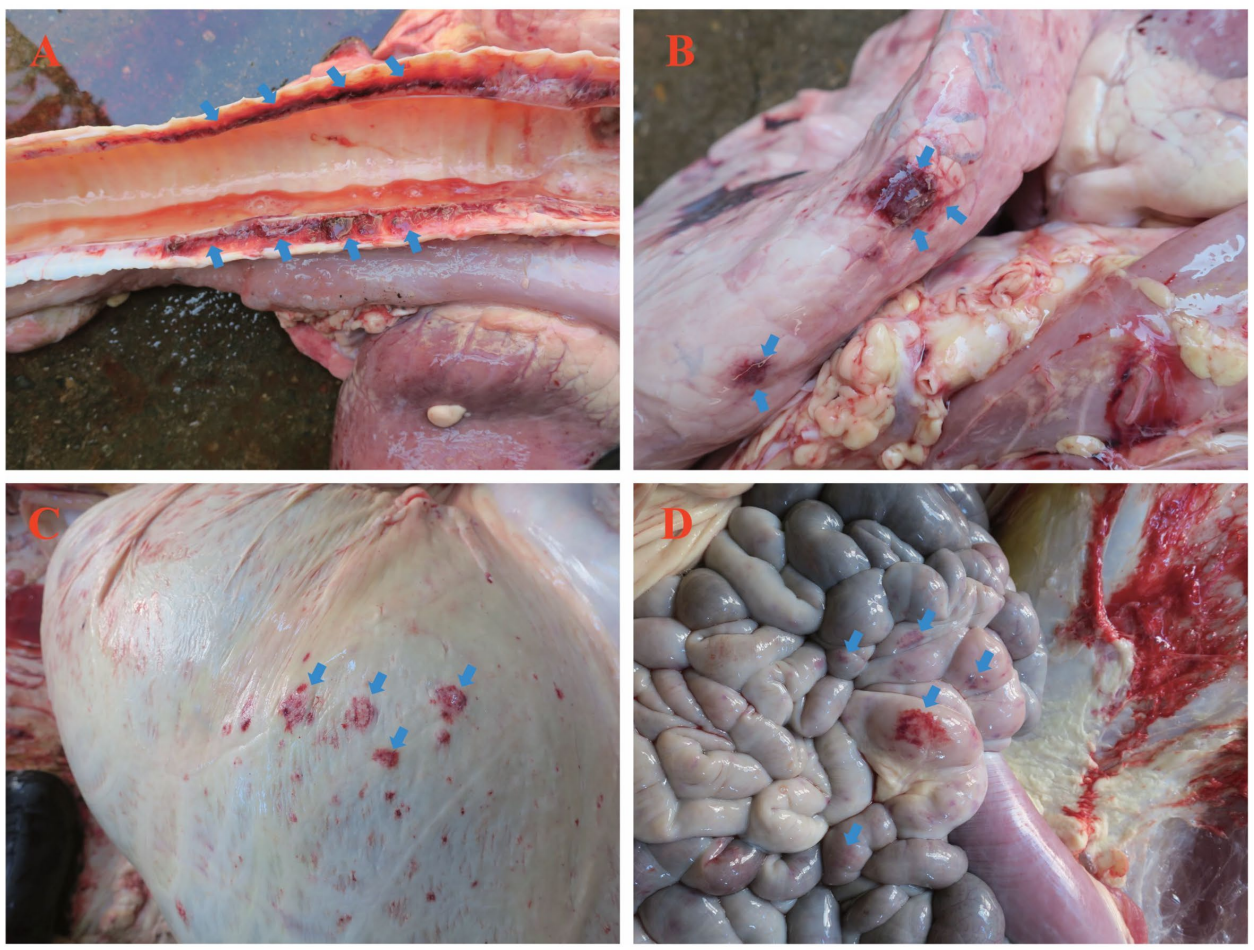
Postmortem findings in yaks, fatally infected with LSDV. **(A)** Hemorrhage in tracheal longitudinal sections; **(B)** Near-circular hemorrhagic plaques on the pulmonary pleura; **(C)** Pinpoint or patchy hemorrhage on the ruminal serosa; **(D)** Hemorrhage in the jejunal serosa. Blue arrows indicate hemorrhagic foci.

Postmortem examination revealed lesions predominantly in the respiratory and digestive systems. The trachea exhibited congestion, hemorrhage, and excessive mucus (Figure 2A). The lungs displayed patchy hemorrhagic foci protruding from the serosal surface (Figure 2B). The rumen serosa showed multifocal hemorrhagic nodules resembling pox lesions (Figure 2C). Hemorrhage was also observed in the jejunal serosa, mirroring ruminal findings (Figure 2D).

### 3.3 Histopathology and immunohistochemistry

**Figure 3:**
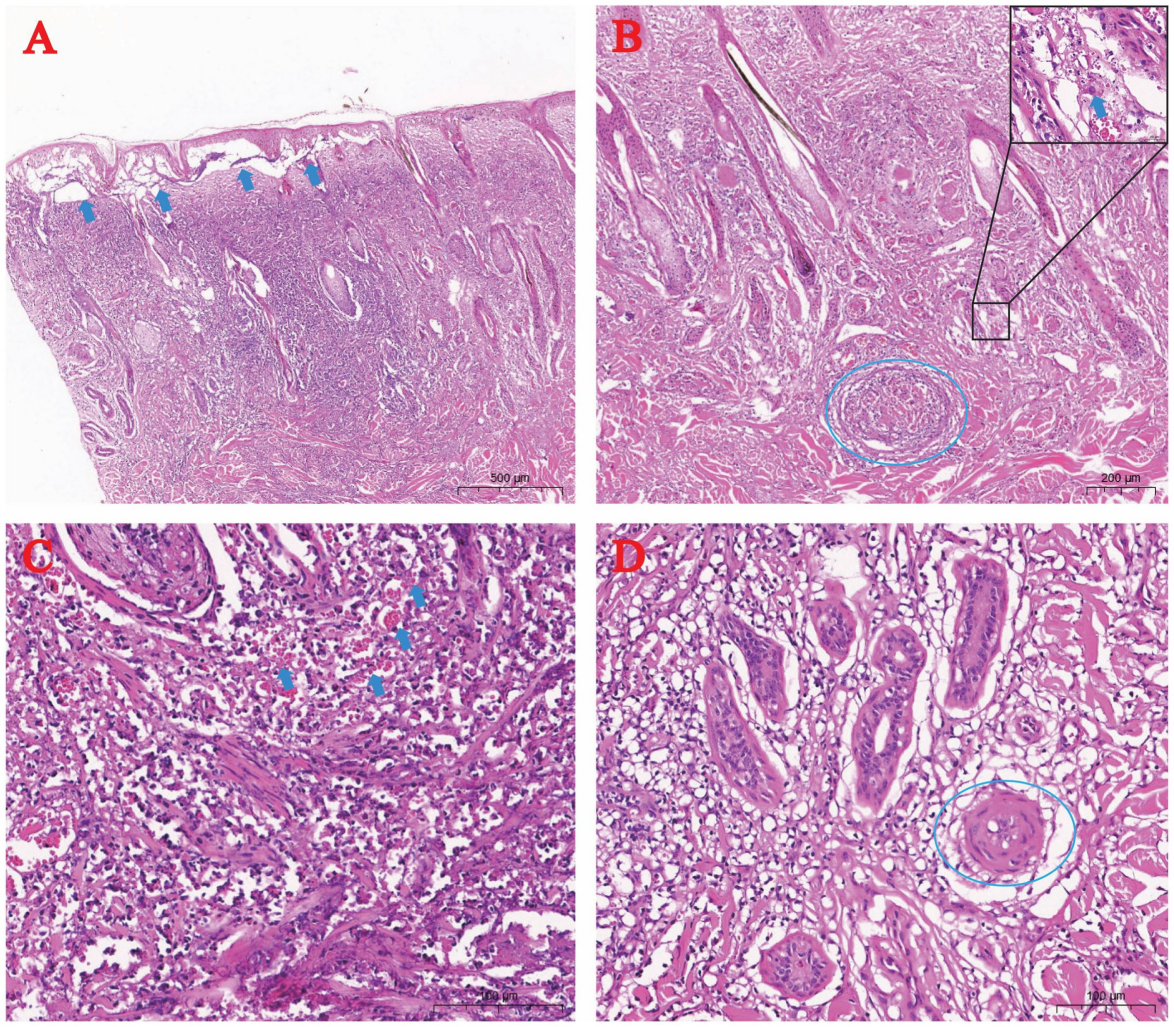
Histopathology of skin pox lesions in naturally LSDV-infected yaks (H&E staining). **(A)** Epidermal-dermal edema (arrow); **(B)** Dermal vasculitis (dashed circle) with rare cytoplasmic inclusion bodies (arrow); **(C)** Lymphocytic and erythrocytic infiltration in the dermis (arrow); **(D)** Arteriolar vasculitis in the dermis (dashed circle).

H&E staining identified epidermal-dermal edema with disrupted rete ridge architecture (Figure 3A). Severe vasculitis in the dermis featured perivascular lymphocyte infiltration, vascular occlusion, and eosinophilic viral inclusion bodies in affected keratinocytes (Figure 3B). Mid-dermal layers showed extensive lymphocytic and erythrocyte infiltration (Figure 3C). Deep dermal regions exhibited marked arteriolar vasculitis, endothelial necrosis, and luminal obstruction (Figure 3D).

**Figure 4:**
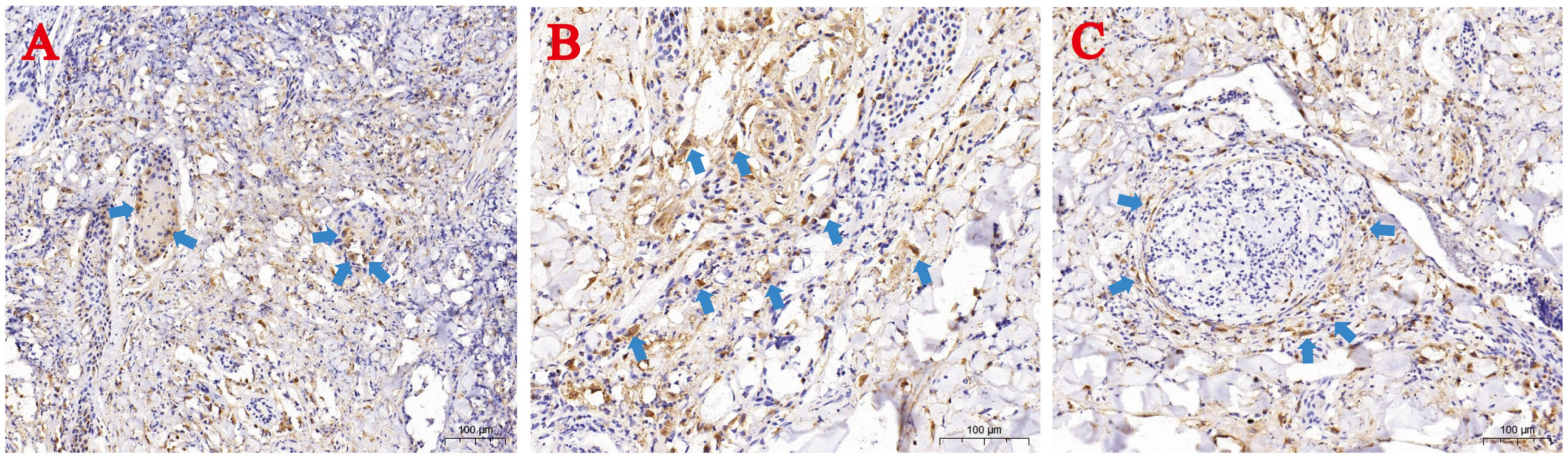
Immunohistochemical (IHC) staining of skin lesions from naturally LSDV-infected yaks. **(A)** IHC-positive hair follicle epithelial cells; **(B)** Deep dermal macrophages; **(C)** Perivascular lymphocytes. Blue arrows highlight IHC-positive cells.

Immunohistochemistry using anti-LSDV126 antibody highlighted viral antigen (brown staining) in hair follicle epithelial cells (Figure 4A), deep dermal macrophages (Figure 4B), and perivascular lymphocytes (Figure 4C).

### 3.4 Phylogenetic analysis

**Figure 5:**
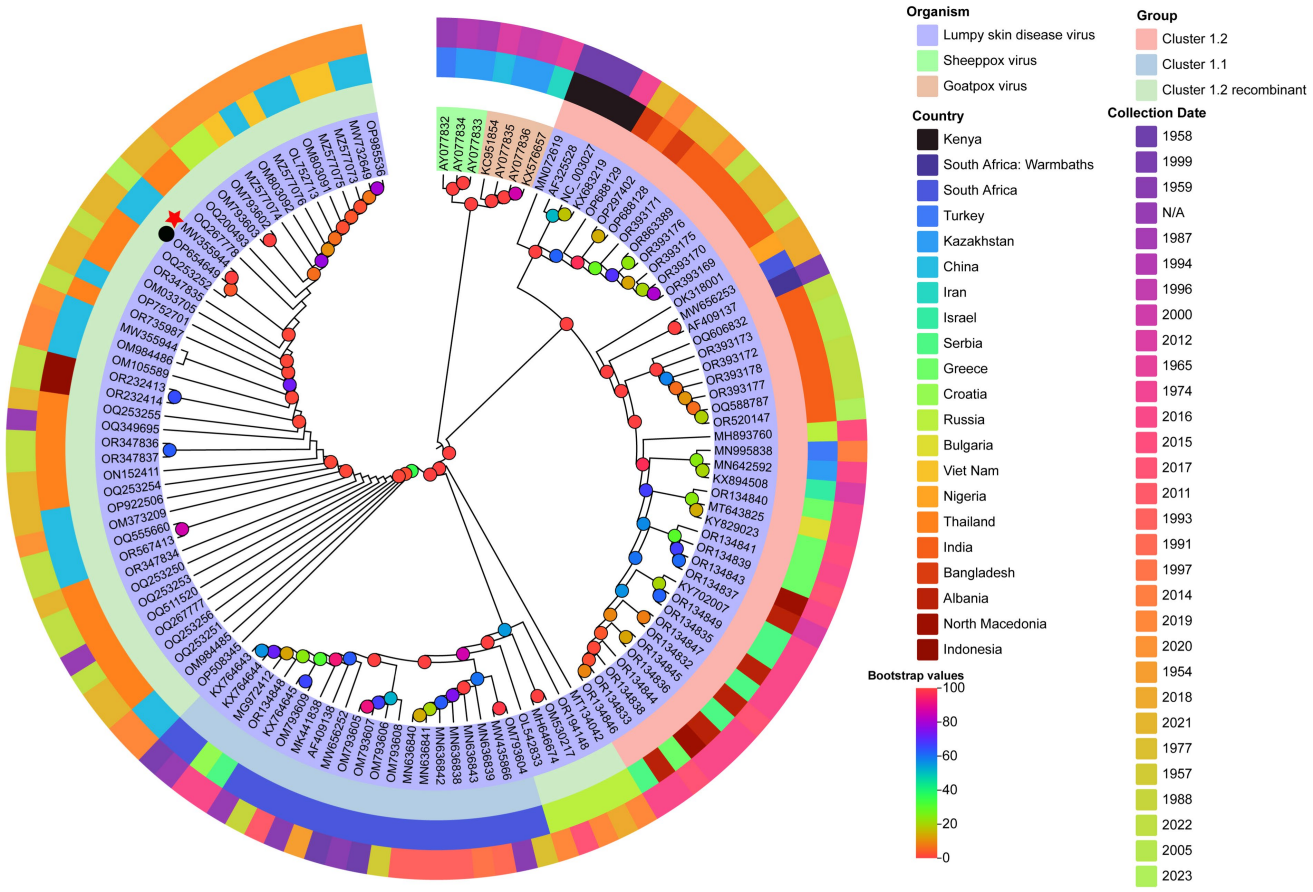
Maximum likelihood phylogenetic tree of LSDV based on full-genome sequences. The tree was constructed using PhyloSuite v1.2.3 (38) with IQ-TREE v2.2.0 (41), rooted with Sheeppox virus and Goatpox virus as outgroups. The best-fit substitution model (TVM + F + I + I + R2) was selected by BIC. Bootstrap values are color-coded at branch nodes. GenBank accession numbers, host species (color-coded), and LSDV sublineages (innermost ring) are annotated. The middle ring denotes country of origin, and the outermost ring indicates collection year. Red stars and black circles mark yak-derived LSDV genomes from this study and prior work, respectively.

Maximum likelihood phylogenetic trees constructed from full-genome sequences of LSDV, SPPV, and GTPV (NCBI database) revealed three major LSDV clades: Cluster 1.1, Cluster 1.2, and Cluster 1.2 recombinant (Figure 5). Cluster 1.1 included strains from South Africa (1954–2016) and Balkan countries. Cluster 1.2 comprised strains from Africa, the Balkans, the Middle East, Eastern Europe, and South/Central Asia (1958–2023). Cluster 1.2 recombinant subclade contained strains from East/Southeast Asia and Asian Russia (2017–2023). The yak-derived LSDV strain in this study (LSDV/China/GS/Yak, GB No. MW355944.1) clustered within the Cluster 1.2 recombinant subclade, consistent with Chinese, Thai, and Russian recombinant strains (19).

**Figure 6:**
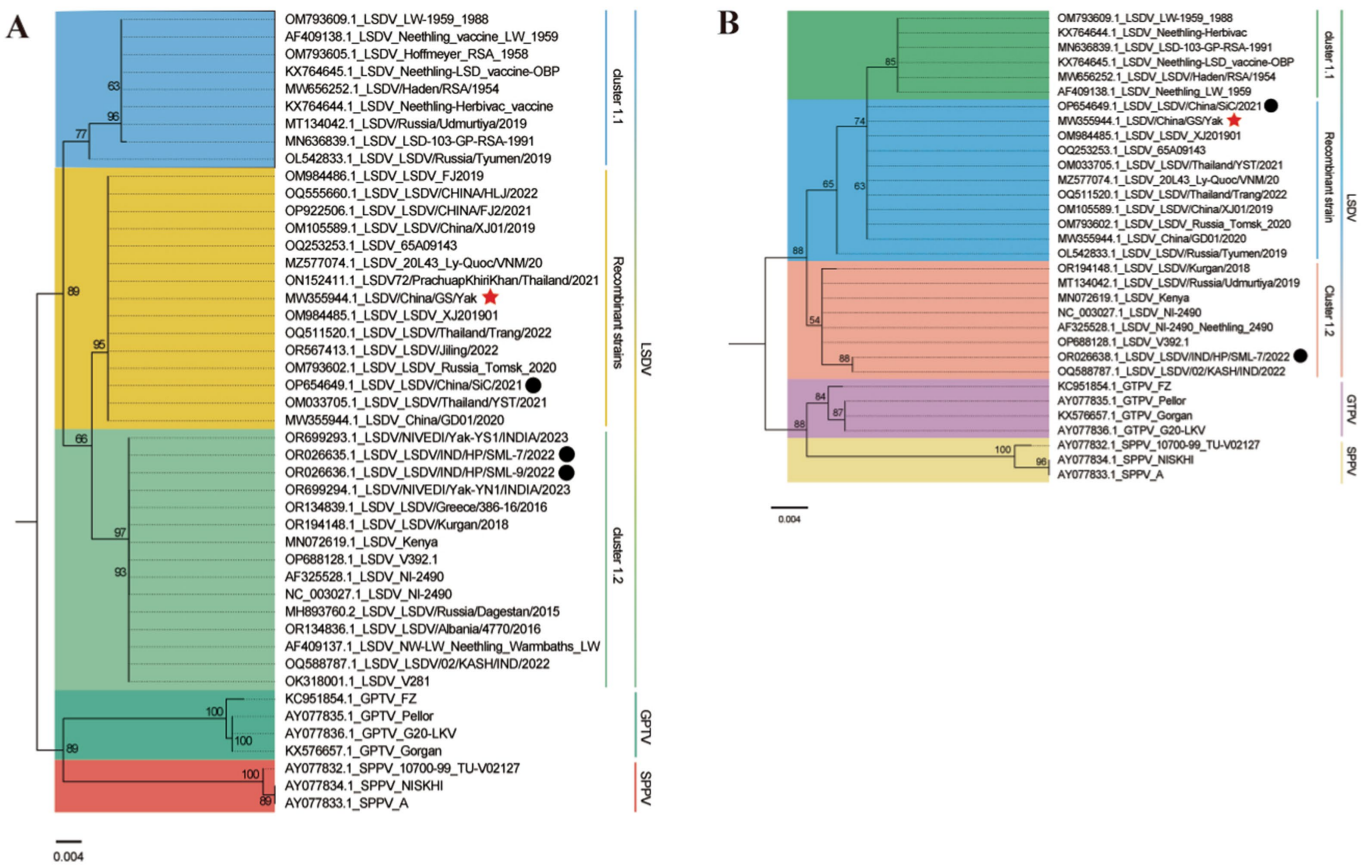
Maximum likelihood phylogenetic trees based on LSDV GPCR **(A)** and RPO30 **(B)** gene sequences. Trees were generated using PhyloSuite v1.2.3 (38) with IQ-TREE v2.2.0 (41), rooted with Sheeppox virus and Goatpox virus. Substitution models (K3Pu + F + I for GPCR; HKY + F + I for RPO30) were selected by BIC. Bootstrap values <50 are omitted. Branch labels include strain metadata, with colors denoting host species or LSDV subtypes. Red stars and black circles mark yak-derived LSDV sequences from this study and previous work.

In contrast, Indian yak LSDV strains formed a distinct Cluster 1.2 non-recombinant branch (43). GPCR and RPO30 gene-based phylogenies corroborated these findings, with LSDV/China/GS/Yak grouping within the recombinant subclade (Figure 6).

## 4 Discussion

Recombinant LSDV infections in yaks have been reported in China’s Sichuan Province and India’s Himachal Pradesh near the Qinghai-Tibet Plateau (19, 43). Our study demonstrates active LSDV transmission among yaks in this region, supported by severe clinical manifestations (fever, hemorrhagic skin nodules, lymphadenopathy) and a 100% viral DNA detection rate in skin lesions. The high viral load in skin samples suggests potential environmental persistence via scab shedding critical concern given yaks’ year-round outdoor grazing practices (44). Concurrent respiratory and gastrointestinal lesions (tracheal hemorrhage, pulmonary necrosis, ruminal hemorrhagic plaques) indicate systemic viral spread, likely exacerbated by high-altitude hypoxia, contributing to the observed 46.67% mortality. Of particular concern is the adaptation of the virus to cold climates (up to 3,600 m elevation in this study), raising alarms about its potential invasion into ecologically fragile core yak habitats, which host over 90% of the world’s 16 million yaks (45, 46). Traditional migratory grazing and limited veterinary infrastructure may further accelerate silent transmission.

Phylogenetic analysis revealed a striking geographic dichotomy in LSDV evolution. Classical non-recombinant Cluster 1.2 strains dominate in Bangladesh (26), India (27), Nepal (28), and Myanmar (29), whereas the yak-derived strain in this study (LSDV/China/GS/Yak) clusters within the emerging recombinant Cluster 1.2 sub-lineage (Neethling vaccine-like recombinants) circulating in China, Russia, and Thailand since 2017. The dominance of this recombinant lineage across East Asia suggests independent evolutionary trajectories or differential vaccine pressures. Notably, GPCR/RPO30 gene homology with strains from Russia (47) and Mongolia (24) implies undocumented cross-border transmission via livestock movement along the Silk Road Economic Belt. The rapid replacement of ancestral strains by recombinant strains within approximately 6 years highlights the need to investigate potential adaptive advantages, such as immune evasion or changes in vector competence.

Histopathological findings underscore the heightened pathogenicity of recombinant Cluster 1.2 LSDV in yaks. Severe vasculitis with endothelial necrosis and trans-mural lymphocyte infiltration mirrors poxvirus-endothelial interactions in human smallpox, raising questions about potential zoonotic barriers (48, 49). Viral tropism for hair follicle epithelium may enhance environmental persistence via keratinocyte shedding—a key adaptation for survival in high-UV plateau environments.

While LSD morbidity typically ranges from 3 to 85% (rarely reaching 100%) with mortality below 10% (4, 8, 21), Classical Cluster 1.2 strains in India caused mild, non-fatal infections in yaks (43). In contrast, our study and prior work (19) report 46.67–53.33% mortality in yaks infected with recombinant Cluster 1.2 LSDV, highlighting heightened susceptibility to emerging recombinants. Given yaks’ grazing patterns and limited veterinary resources, unchecked spread of recombinant strains across the Qinghai-Tibet Plateau could precipitate catastrophic losses.

## 5 Conclusion

LSDV represents an emerging threat to yaks in the Qinghai-Tibet Plateau, demonstrating exceptional virulence with a 46.67% case fatality rate. Histopathological evidence reveals dual pathogenic mechanisms: microcirculatory disruption and direct cytopathic effects. Phylogenetically, Chinese yak LSDV strains cluster within the recombinant 1.2 sublineage, showing 99.2–99.4% homology with East Asian and Russian variants. Notably, this contrasts with non-recombinant Indian yak strains (Cluster 1.1), revealing divergent evolutionary paths between South Asian and East Asian lineages.

## Data Availability

The datasets presented in this study can be found in online repositories. The names of the repository/repositories and accession number(s) can be found at: https://www.ncbi.nlm.nih.gov/genbank/, MW355944.

## Footnotes

^1^ https://www.3dhistech.com/research/software-downloads/

^2^ https://www.dnastar.com/software/lasergene/

^3^ http://www.ncbi.nlm.nih.gov

^4^https://www.chiplot.online/tvbot.html; accessed on December 1, 2024.
